# Role of hippocampal circKcnk9 in visceral hypersensitivity and anxiety comorbidity of irritable bowel syndrome

**DOI:** 10.3389/fncel.2022.1010107

**Published:** 2022-11-17

**Authors:** Yuan Liu, Zhong Chen, Wei Lin, Yifei Zhou, Zihan Liu, Ruixia Zhao, Yu Chen, Bin Wu, Aiqin Chen, Chun Lin

**Affiliations:** ^1^Fujian Provincial Key Laboratory of Brain Aging and Neurodegenerative Diseases, Pain Research Institute, School of Basic Medical Sciences, Fujian Medical University, Fuzhou, China; ^2^Department of Pediatrics, The First Affiliated Hospital of Fujian Medical University, Fuzhou, China

**Keywords:** circkcnk9, miR-124-3p, EZH2, hippocampus, irritable bowel syndrome

## Abstract

Irritable bowel syndrome (IBS) is a common gastrointestinal disorder characterized by recurrent visceral pain and altered bowel habits (diarrhea or constipation). However, the molecular and pathological mechanisms are poorly understood. This study found neonatal colorectal distension to induce visceral hypersensitivity and anxiety. The expression of hippocampal circKcnk9, a novel circRNA, was significantly increased in IBS-like rats. Interestingly, CA1 shcircKcnk9 treatment inhibited long-term potentiation (LTP) and alleviated visceral hypersensitivity and anxiety in IBS-like rats, whereas overexpression of CA1 circKcnk9 induced LTP, visceral hypersensitivity, and anxiety in controls. Several experiments indicated that increased CA1 circKcnk9 acted as a miR-124-3p sponge, which resulted in the inhibitory effect of miR-124-3p on gene silencing. There was a negative correlation between circKcnk9 and miR-124-3p expression. As expected, CA1 administration of agomiR-124-3p decreased CA1 LTP, visceral hypersensitivity, and anxiety in the IBS-like rats. In contrast, CA1 treatment with antagomiR-124-3p induced LTP, visceral hypersensitivity, and anxiety in the controls. Furthermore, bioinformatic analysis and experimental data showed that EZH2 is a circKcnk9/miR-124-3p target gene, and increased EZH2 expression was involved in visceral hypersensitivity and anxiety in IBS-like rats by enhancing hippocampal synaptic plasticity. In conclusion, early life stress induces increased expression of circKcnk9 in the CA1 of IBS-like rats. Increased circKcnk9 expression regulates synaptic transmission and enhances LTP, leading to visceral hypersensitivity and anxiety in IBS-like rats. The underlying circKcnk9 signaling pathway is miR124-3p/EZH2. Increased circKcnk9 reinforces its sponging of miR124-3p and strongly suppresses miR124-3p activity, resulting in increased expression of the target gene EZH2. This study provides a new epigenetic mechanism for visceral hypersensitivity and anxiety in IBS-like rats.

## Introduction

Irritable bowel syndrome (IBS) is characterized by recurrent episodes of abdominal pain and irregular bowel habits (diarrhea or constipation) without recognizable organic pathological changes (Ma C. et al., 2021). Visceral hypersensitivity might be responsible for the clinical symptoms (Poulsen et al., 2017). Increased visceral sensitivity is observed in up to 60% of patients with IBS (Mujagic et al., 2017). Patients with IBS often suffer from negative emotional comorbidities, such as anxiety and depression, due to persistent visceral hypersensitivity (Black et al., 2021; Midenfjord et al., 2021; Yu et al., 2021). Chronic exposure to adverse life events increases personal susceptibility to IBS disorders, especially in the early stages (O'Mahony et al., 2017). However, the molecular mechanisms involved in visceral hypersensitivity and emotional comorbidity in IBS remain unclear.

Long-lasting phenotypes, such as chronic pain and emotional comorbidity, may be associated with epigenetic modulation of gene expression (Louwies et al., 2019). Circular RNAs (circRNAs) are post-transcriptional regulatory molecules. CircRNAs are a type of non-coding RNA formed by a closed-loop structure without the 5' hat and 3' polyadenylated tail. Ample evidence suggests that dysregulation of circRNAs is implicated in many diseases, including cancer (Gao X. et al., 2021; Chen et al., 2022), chronic pain (Zheng et al., 2022), and emotional comorbidity (Huang et al., 2020). Different circRNAs are involved in different types of chronic pain. CircAnks1a regulates hypersensitivity in rodent models of neurological pain (Zhang S. B. et al., 2019). A cancer pain study found that circStrn3 regulates bone cancer pain in rats (Zhang et al., 2020). Recent data also supports the regulation of circRNAs in negative emotions. In addition, CircSTAG1 was significantly decreased in the hippocampus of chronically unpredictable stress-treated mice and the peripheral blood of patients with major depressive disorder (Huang et al., 2020). Visceral hypersensitivity and negative emotions are often comorbid with IBS. Our previous study demonstrated that long-term potentiation (LTP) was enhanced in the hippocampal CA1 region of IBS-like rats (Chen et al., 2014), indicating that early life stress results in abnormal synaptic plasticity and adverse memory in the hippocampus. The hippocampus is associated with memory formation, visceral function, and emotional modulation (Ren et al., 2022). Several studies have suggested that hippocampal circRNAs are involved in pain regulation (Mao et al., 2020), negative emotions (Huang et al., 2020), and synaptic plasticity (Xu et al., 2020). We hypothesized that early life stress-induced abnormal expression of hippocampal circRNA, which regulates synaptic plasticity, consequently leads to visceral hypersensitivity and anxiety comorbidity in IBS-like rats. We found that the expression of hippocampal circKcnk9, a novel circRNA, was significantly increased in IBS-like rats. Studies have reported that circRNAs can act as miRNA sponges to modulate the expression of target genes (Dube et al., 2019; Zeng et al., 2021). Mice exposed to chronic ultra-mild stress exhibit increased depression-like behaviors and reduced hippocampal expression of brain-enriched miR-124 (Higuchi et al., 2016). In addition, miR-124-3p is involved in neuropathic pain in chronic constriction injury (CCI) rat models (Grace et al., 2018). Furthermore, we used the miRanda database to predict the binding sites and found that circKcnk9 had three miR-124-3p binding sites. Literature and bioinformatic results indicate that miR-124-3p may act as a miR-124-3p sponge in IBS-like rats. However, further experiments are needed to elucidate this.

In this study, we first determined the role of hippocampal circKcnk9 in IBS-like rats with visceral hypersensitivity and anxiety. Next, we investigated whether circKcnk9 plays a regulatory role as a miR-124-3p sponge. Finally, we explored the downstream target genes of circKcnk9 and miR-124-3p using various experimental methods. This study could provide a new epigenetic mechanism for IBS-like rats with visceral hypersensitivity and anxiety.

## Materials and methods

### Animals

Neonatal male Sprague-Dawley rats were obtained from the Department of Experimental Animal Center of Fujian Medical University. The animals were maintained in a specific pathogen-free-grade environment at the animal center. Weaning is typically initiated 21 days after birth. The experiments were approved by the Animal Care and Use Committee of Fujian Medical University. IBS-like rats were established by 60 mmHg colorectal distension (CRD) stimulation once daily for 1 min during postnatal days 7–14 (Al-Chaer et al., 2000). We examined the electromyographic (EMG) magnitude in response to graded strengths of CRD pressures in IBS-like and control rats at 6–8 weeks to assess visceral sensitivity.

### Stereotactic cannulation surgery and infusion

The rats were anesthetized with isoflurane (2%) and fixed on a stereotactic instrument (Ruiwode Life Science, China). Following routine skin sterilization, a midline scalp incision was made, and local anesthesia was administered with lidocaine. The skull was exposed and cleaned by scraping it with 10% hydrogen peroxide. All surgical procedures were performed under aseptic conditions, and no infection was detected.

The coordinates of the injection locations were centered at 4.0 mm in the anteroposterior plane, 2.5 mm in the mediolateral plane, and 2.8 mm in the dorsoventral plane. The rats underwent stereotaxic surgery for double cannula (inner radius of 2.8 mm, inner diameter: 0.3 mm, outer diameter: 0.48 mm, Ruiwode Life Science, China) implantation and were allowed to rest for at least seven days. Continuous intrahippocampal administration (agomiR-124-3p/nc and siEZH2 for IBS-like rats; antagomiR-124-3p/nc for controls) was administered once daily for 3 days.

IBS-like rats that received an intrahippocampal injection of LV-hSyn-mcherry-5'MiR-30a-shcirc7685-3'MiR-30a-WPRE (shcircKcnk9, 1μL, Supplementary Figure S1, BrainVTA, Wuhan) were allowed to rest for 10 days postoperatively. The control rats received an intrahippocampal injection of aav-Kcnk9 virus (PFD-rAAV-hSyn-circ7685-nEF1a -EGFP-hGH, 1μL, Supplementary Figure S1, BrainVTA, Wuhan). They were allowed to rest for 21 days postoperatively (the timeline of this process is correlated with that of viral production).

### Behavioral tests

All the experiments were randomized. All behavioral tests were conducted between 13:00 and 16:00. During the experiment, the experimental environment was kept noise-free, and only 6–8-week-old male rats were used for behavioral assays. All animals were allowed to habituate for 1 h before behavioral testing. The videos were recorded and analyzed using video-tracking software (Yishu, Shanghai, China).

#### Open field test

The open field test (OFT) was performed in the novel environment, which was a black Plexiglas area (100 × 100 × 60 cm). The equipment was wiped with 10 % ethanol three times to eliminate odor clues between each rat. The rats were placed in the center of the Plexiglas area and allowed to freely explore the field for 5 min. The total travel distance, travel distance in the central area, and time spent in the central area were measured as anxiety indicators. The rat's body tracks were recorded by the Yishu Vision tracking system (Shanghai, China).

#### Elevated plus maze test

The elevated plus maze (EPM) test consisted of four arms: two open and two closed arms (LWH 500 × 100 × 450 mm). At the beginning of the experiment, the rats were individually placed at the junction of the four arms. The equipment was wiped with 10% ethanol three times to eliminate odor clues between each rat. The tracking of the rats was recorded for 5 min using video-tracking software and saved for future analysis on a computer. Additionally, the number of open-arm entries, open-arm time, and open-arm distance in EPM was recorded for the indicators of anxiety-like behavior. The video-tracking system was the same as that of the OFT analysis system.

#### Electromyography

Electromyography (EMG) was performed to assess visceral hypersensitivity. Rats (6–8 weeks old) were anesthetized with isoflurane. Before the CRD procedure, a glycerol-lubricated balloon was inserted into the rectum. A pair of bipolar electrodes were implanted in shallow anesthetized rats' abdominal external oblique musculature to detect EMG activity. Under isoflurane superficial anesthesia, the discharge of the rat ventral oblique muscle was recorded at CRD pressures of 40 and 60 mmHg. The EMG responses to different degrees of CRD were recorded using the RM6240BD system (Chengdu, China). Data were analyzed by averaging the baseline amplitudes. Values over the baseline were used to assess visceral hypersensitivity (Fan et al., 2020).

#### Morris water maze

The Morris water maze (MWM) consisted of a circular tank circled with dark curtains and was used to assess hippocampal-dependent spatial learning and memory. The water was made opaque by adding the prepared Chinese ink and separated into four equal quadrants. The first day of water maze training was dedicated to adapting rats to the aquatic labyrinth. In the center of the fourth quadrant, a hidden platform was located 1 cm below the water's surface. The trials were performed four times per day for a total of 6 days, and the rats' latency in finding the platform was recorded (Levit et al., 2021). The rat's body trajectories were recorded using an animal visual tracking system (YiShu, Shanghai, China).

### Protein extraction and western blot

A radioimmunoprecipitation assay (RIPA) buffer was obtained from Millipore. A protease inhibitor (PMSF) was purchased from Sigma-Aldrich. Proteins from the hippocampus of rats were extracted using RIPA and PMSF, separated by 8% SDS-PAGE, and electro-transferred onto PVDF (Invitrogen, USA) membranes, which were probed with rabbit anti-enhancer of zeste homolog 2 (EZH2) (5246S, 1:1000, Cell Signaling Technology [CST], USA), rabbit anti-EED (85322, 1:1000, CST, USA), rabbit anti-SUZ12 (3737, 1:1000, CST, USA), and mouse anti-β-actin primary antibody (8226, 1:1000, Abcam, USA). Furthermore, protein expression levels and quantification of IF were detected using ImageJ (http://rsb.info.nih.gov/ij/).

### RNA extraction and qPCR

According to the manufacturer's instructions, total RNA was extracted using a TRIzol reagent (Invitrogen, USA). An Evo-M-MLV reverse transcription kit (Accurate Biology, China) was used to perform reverse transcription of circRNA and mRNA. The miRNA 1st-strand cDNA synthesis kit (Accurate Biology, China) was used to perform reverse transcription for microRNA. After reverse transcription, qRT-PCR was performed. The primer sequences are shown in Table 1.

**Table 1:** Primer used qRT-PCR detection.

**Gene**	**Primer**	**Sequence (5'−3')**
CIRCKcnk9	Forward	AAAACCACAGGCTGCACATC
	Reverse	CATAACCAGCGTCAGAGGGA
circStk39	Forward	GTGAGAGGCTATGACTTCAA
	Reverse	GCAGATAGTCTAATCCTTCC
circRab30	Forward	CCAACAGAGAGCAGAAGAGT
	Reverse	GGAGCGAAATCTCTCTTGAC
CircQrich1	Forward	ACATGAAGTTCTGAAGGACG
	Reverse	GTACTCTTCAAATGAGATGG
circCept1	Forward	GAAGAGTACCCTCATGGATT
	Reverse	GTTTCCTTGTTGACCGATGC
circTtll5	Forward	GTGAGTTGTGATGATCCAGA
	Reverse	GTAACGGGAGACCAAGATGT
circAff4	Forward	CATGGAGGATCTCATCAGAG
	Reverse	CTGAATTTCCTGATTCCGCC
circZfp827	Forward	CCAGTCTGTCATTTTCCCCA
	Reverse	CTTGACACTGCAGTGAGTCT
mkcnk9	Forward	CGCAAGTCCATCTAAGTGTG
	Reverse	GCATAGAACATACAGAAGGCC
mEZH2	Forward	TAAGGGCACA
	Reverse	TACATTCAGG
U6	Forward	GGAACGATACAGAGAAGATTAGC
	Reverse	TGGAACGCTTCACGAATTTGCG
GAPDH	Forward	ACTCCCATTCTTCCACCTTTG
	Reverse	CCCTGTTGCTGTAGCCATATT
miR-124-3p		AGTGCAGGGTCCGAGGTATT

### RNA immunoprecipitation

The hippocampi were fragmented using ultrasonography. RNA immunoprecipitation was performed using the RNA immunoprecipitation (RIP) RNA-Binding Protein Immunoprecipitation Kit (BersinBio Biotech, Guangzhou, Guangdong, China) (Ma S. et al., 2021) with anti-AGO2 (#2897, 1:150, CST, USA). The input was set as a positive control, and IgG was used as a negative control. Levels of coprecipitated circKcnk9 and miR-124-3p were evaluated using quantitative real-time PCR (qPCR).

### Luciferase reporter assay

PC12 cells were cultured in Dulbecco's Modified Eagle Medium (DMEM) containing 5% FBS (ThermoFisher, USA) and 1% penicillin/streptomycin and were transfected with mimic124-3p/nc at a concentration of 50 nM oligonucleotides using Lipofectamine 3000 (Invitrogen), according to the manufacturer's protocol. For the luciferase reporter assay, pmirGLO dual-luciferase vectors (GenePharma, Shanghai, China) were used to construct dual-luciferase reporter plasmids. The sequences of miR-124-3p and circKcnk9 were separately cloned into vectors (Supplementary Figure S2). PC12 cells were co-transfected with wild-type pmirGLO-circKcnk9 or mutated type and miR-124 mimics (negative control). After 48 h induction, the luciferase activity was assessed using a dual-luciferase reporter kit (Promega, Madison, WI, USA). GloMax^®^ 20/ 20 system (Promega, USA) was used to test luciferase activity. The relative firefly luciferase activity was normalized to Renilla luciferase activity.

### RNA fluorescent in situ hybridization

Rats were deeply anesthetized with Ulatan (0.5 mL/100 g) and transcardially perfused with 500 mL of ice-cold 0.9% NaCl, followed by 750 mL of 4% paraformaldehyde. The brains were removed from the skull and placed in 4% paraformaldehyde overnight. After gradient dehydration with 20% and 30% sucrose (Sigma, USA), tissues were embedded with an optimal cutting temperature (OCT) compound and sectioned using a cryotome (Leica, Germany). RNA localization and quantification were determined using a Fluorescence *in situ* Hybridization (FISH) kit (GenePharma, Shanghai, China) and an RNA probe from Exiqon (Exiqon Life Sciences, Denmark), according to the manufacturer's protocol. For combined RNA FISH and immunostaining, we first performed RNA FISH, followed by immunofluorescence.

RNA degradation was ruled out in all the steps by using diethyl pyrocarbonate-treated water.

### Immunofluorescence

The sections (20–40 μm) were removed from the −20°C refrigerator for half an hour before use. Phosphate buffer solution (PBS) was used to wash out the OCT. A Pap pen was used to circle the tissues. The sections were blocked with an immunostaining blocking buffer solution containing 5% goat serum and 0.3% Triton^TM^X-100 at room temperature for 2 h (or 37°C for 30 min) and then incubated with primary antibodies at 4°C for 24–48 h. Sections were incubated with secondary antibodies at room temperature for 2 h before being washed with PBS (three times for 15 min). Immunofluorescence analysis of frozen sections was performed using the primary antibodies described in Table 2.

**Table 2:** Primary antibodies.

**Antibodies**	**Art. No**	**Ratio**
EZH2	CST 5246S	1:100
GFAP	CST 3670S	1:300
NEUN	Millipore MAB377	1:300
IBA-1	Woko PTR2404	1:5000
DAPI	Beyotime C1002	1:1000

Secondary antibodies: Goat anti-Rabbit lgG, 488 (150073, 1:300, Abcam); Goat anti-Mouse lgG, 488 (150105, 1:300, Abcam); Goat anti-Rabbit lgG 594 (8889S, 1:500, CST); Goat anti-Mouse lgG, 594 (150108, 1:300, Abcam).

Immunofluorescence and FISH images were captured using a Leica SP5 confocal microscope equipped with 405, 488, and 594 lasers. The contrast of the final images was adjusted using Photoshop (Adobe Systems, Mountain View, CA, USA).

### Bioinformatic analysis

The delineation of circRNA/miRNA interactions was predicted using miRanda (http://www.microrna.org/microrna/home.do). Circular plots of circRNA-miRNA-binding sites were plotted using (http://www.bioinformatics.com.cn), a free online platform for data analysis and visualization. Venn diagrams were created using the Lianchuan Cloud platform (Hangzhou Lianchuan Biotechnology Co. Ltd., Hangzhou, China). A PPI network of the HUB gene was obtained using the STRING database (https://string-db.org) and visualized using Cytoscape (Shannon et al., 2003).

### Slice preparation and field potential recording

Rats (6–8 weeks) were anesthetized with isoflurane (2%), and the brain was rapidly removed by decapitation. Acute coronal slices (400 μm thick), including the hippocampus, were obtained using a vibrating-knife microtome (Leica VT1000s) in an oxygenated, ice-cold, high-sucrose cutting solution (Sigma, USA). The coronal hippocampal slices were rapidly removed and transferred to 30°C oxygenated (95% O_2_, 5%CO_2_, pH 7.4) artificial cerebrospinal fluid (ACSF). The slices were incubated in an interface-recording chamber maintained at a constant temperature and allowed to equilibrate for at least 1.5 h. Each brain slice was recorded only once.

For recording, the hippocampal slices were transferred to a chamber, submerged, and continuously superfused with ACSF at a flow rate of 1–2 mL/min at room temperature (23°C ± 2°C). The Schaffer collaterals were stimulated, and fEPSPs were recorded from the dendritic layer of the CA1 pyramidal cells, as reported by Kleppisch and colleagues for local field potential recordings in the hippocampus (Kleppisch et al., 1999). Brain slices were stabilized by single electrode stimulation for 10 min. Two episodes of high-frequency stimulation then induced LTP at 10-s intervals.

### Statistical analysis

All data are presented as mean ± standard error of the mean. In behavioral tests and molecular biology experiments, two-tailed independent sample t-tests were performed to determine differences between control and IBS-like rats if the data satisfied the normal distribution. A Wilcoxon correction was performed for two independent samples if the data did not satisfy the normal distribution. Statistical analysis of data from more than two groups was performed using one-way analysis of variance (ANOVA)-LSD-t comparisons if the data were normally distributed. The rank sum test, Kruskal–Wallis H test, and Nemenyi test were performed when the data did not satisfy the normal distribution, and there were more than two groups. In addition, repeated-measures ANOVA was performed to analyze electrophysiological results. The EMG results were analyzed using two-way ANOVA. Correlation analysis was performed using a two-tailed Pearson correlation. Data analysis was performed using GraphPad Prism8 and R4.0.3, and a *P*-value < 0.05 was considered statistically significant.

## Results

### Neonatal CRD induces visceral hypersensitivity and anxiety in rats

**Figure 1:**
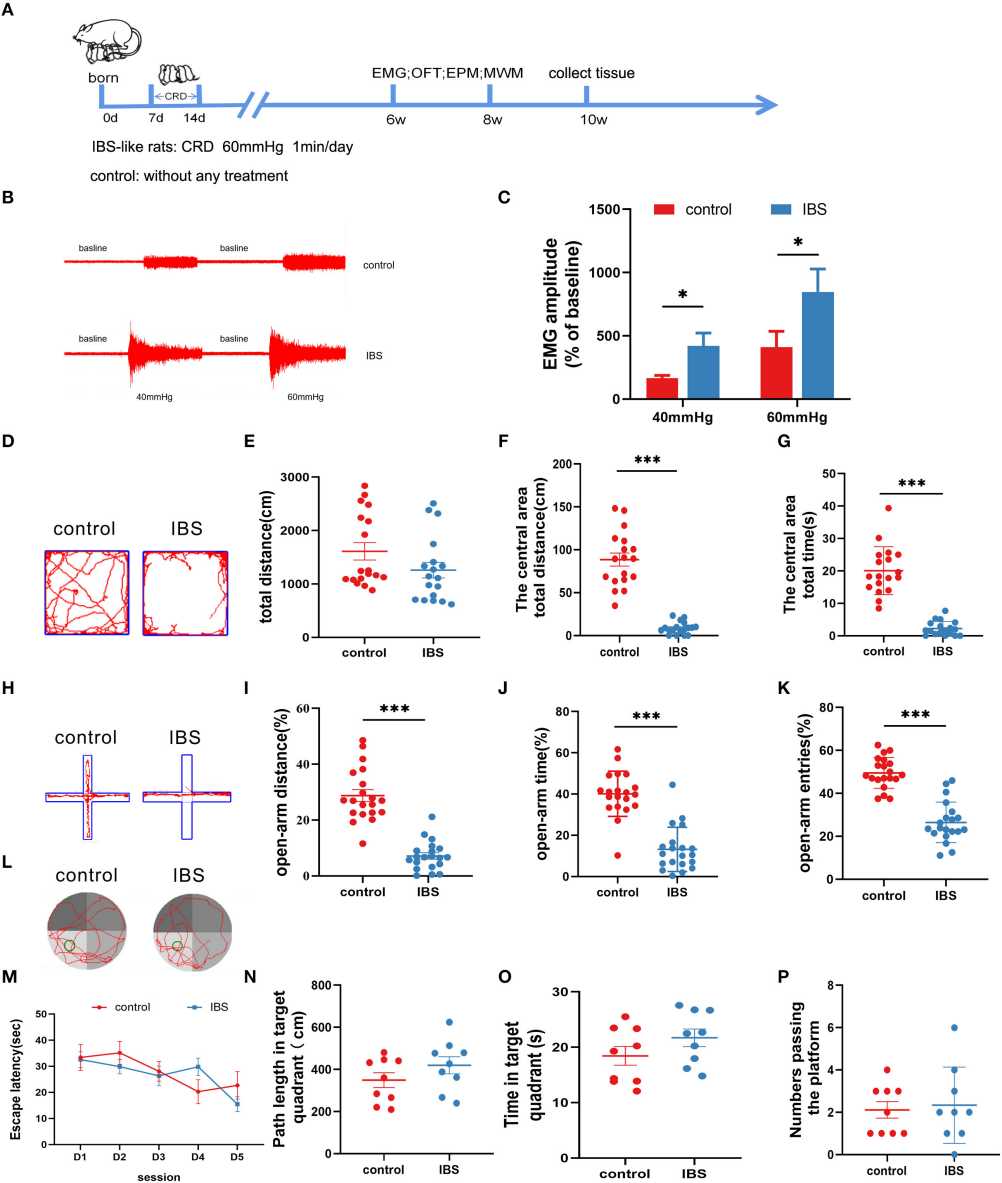
Neonatal CRD induces visceral hypersensitivity and anxiety without cognitive impairment in IBS-like rats. **(A)** Experimental design. **(B,C)** The original graph and statistical chart of EMG amplitude in IBS-like and control rats. *N* = 6, ^*^p < 0.05. **(D)** Original typical traces of OFT. Statistical analysis of the total distance **(E)**, the distance in the central area **(F)**, and the time in the central area **(G)** in the OFT. *N* = 18, ^***^p < 0.001. **(H)** Original typical EPM recordings. Statistical analysis of the distance **(I)**, the time **(J)**, and the number of entries **(K)** in the open arm of EPM. *N* = 20, ^***^p < 0.001. **(L)** The original typical recordings of MWM. The statistical chart of the escape latency **(M)**, path length in target quadrant **(N)**, time in target quadrant **(O)**, and numbers passing the platform **(P)** in MWM. *N* = 9. CRD, colorectal distension; EMG, electromyography; EPM, elevated plus maze test; IBS, irritable bowel syndrome; MWM, Morris water maze; OFT, open field test.

An IBS-like model was established using neonatal CRD (Figure 1A). Visceral sensitivity was assessed by recording the EMG response to the CRD (Figures 1B,C). The EMG amplitudes at 40 and 60 mmHg were significantly increased in IBS-like rats compared to controls. Since patients with IBS are often comorbid with anxiety, we examined anxiety-related behaviors using the OFT and EPM. The OFT results showed that the time and distance in the center area significantly decreased without a difference in the total distance in IBS-like rats (Figures 1D–G). The anxiety-like behavior of IBS-like rats was also observed in the EPM, where the percentage of entries, time, and distance into the open arms significantly decreased (Figures 1H–K). Then, we used the MWM to test whether chronic visceral pain and negative emotions affect cognitive ability. We found no significant difference in escape latency, path length, time of the target quadrant, and number passing the platform between control and IBS-like rats (Figures 1L–P). Taken together, IBS-like rats showed visceral hypersensitivity and comorbid anxiety without cognitive impairment.

### Increased CA1 circKcnk9 is involved in visceral hypersensitivity and anxiety comorbidity by enhancing LTP in IBS-like rats

**Figure 2:**
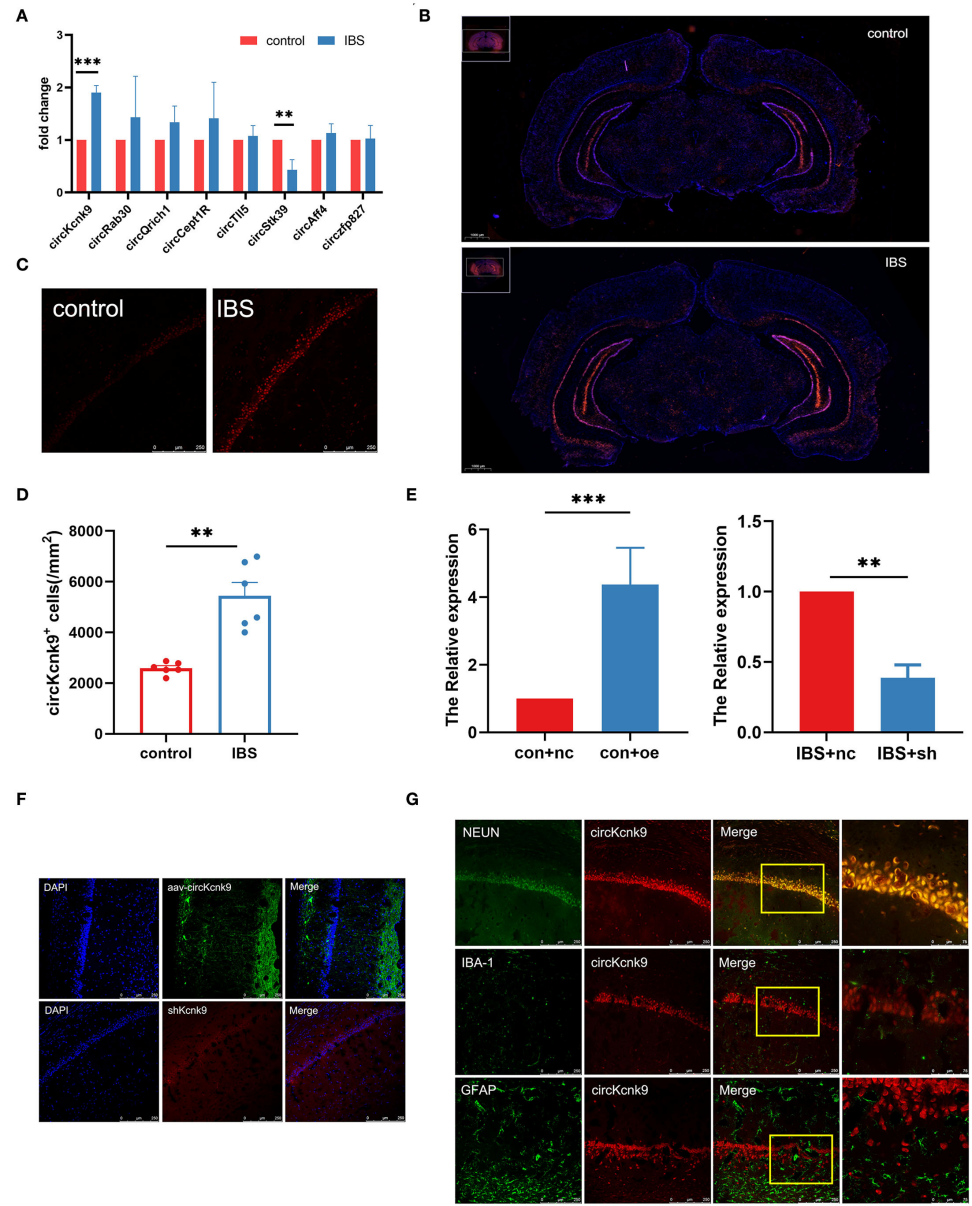
Increased CircKcnk9 is localized in the neurons of hippocampus in IBS-like rats and the expression of circKcnk9 can be modified after aav-circKcnk9 or shcircKcnk9 treatment. **(A)** qPCR quantification of the circular RNA associated with chronic visceral pain. *N* = 6, **p < 0.01, ***p < 0.001. **(B)** Distribution map of circKcnk9 content in brain slices. Scale bar, 1000μm. **(C,D)** Immunofluorescence representative images and quantification of circKcnk9 numbers in the CA1 of IBS-like and control rats. *N* = 6, scale bar, 250μm, **p < 0.01. **(E)** The expression of CA1 circKcnk9 after treatment with aav-circKcnk9 in controls and shcircKcnk9 in IBS-like rats. *N* = 5, **p < 0.01, ***p < 0.001. **(F)** Representative immunofluorescence traces of aav-oe1/shRNA in hippocampal CA1. Scale bar, 250μm. **(G)** Confocal images of CA1 circKcnk9 expression with NEUN, IBA-1 and GFAP. Scale bar, 250μm. IBS, irritable bowel syndrome; DAPI, nucleus staining dye.

First, we used qPCR to examine the expression of pain-related circRNAs in the hippocampus and found a significant increase in circKcnk9 expression (Figure 2A) in IBS-like rats. Next, the distribution of circKcnk9 was determined using FISH. We found that circKcnk9 was highly expressed in the hippocampi of IBS-like rats (Figure 2B). The CA1 region was selected as the quantification zone, and RNA FISH showed that the number of circKcnk9^+^ cells was higher in IBS-like rats than in controls (Figures 2C,D). Furthermore, circKcnk9 was largely colocalized with neurons, not microglia or astrocytes (Figure 2G).

**Figure 3:**
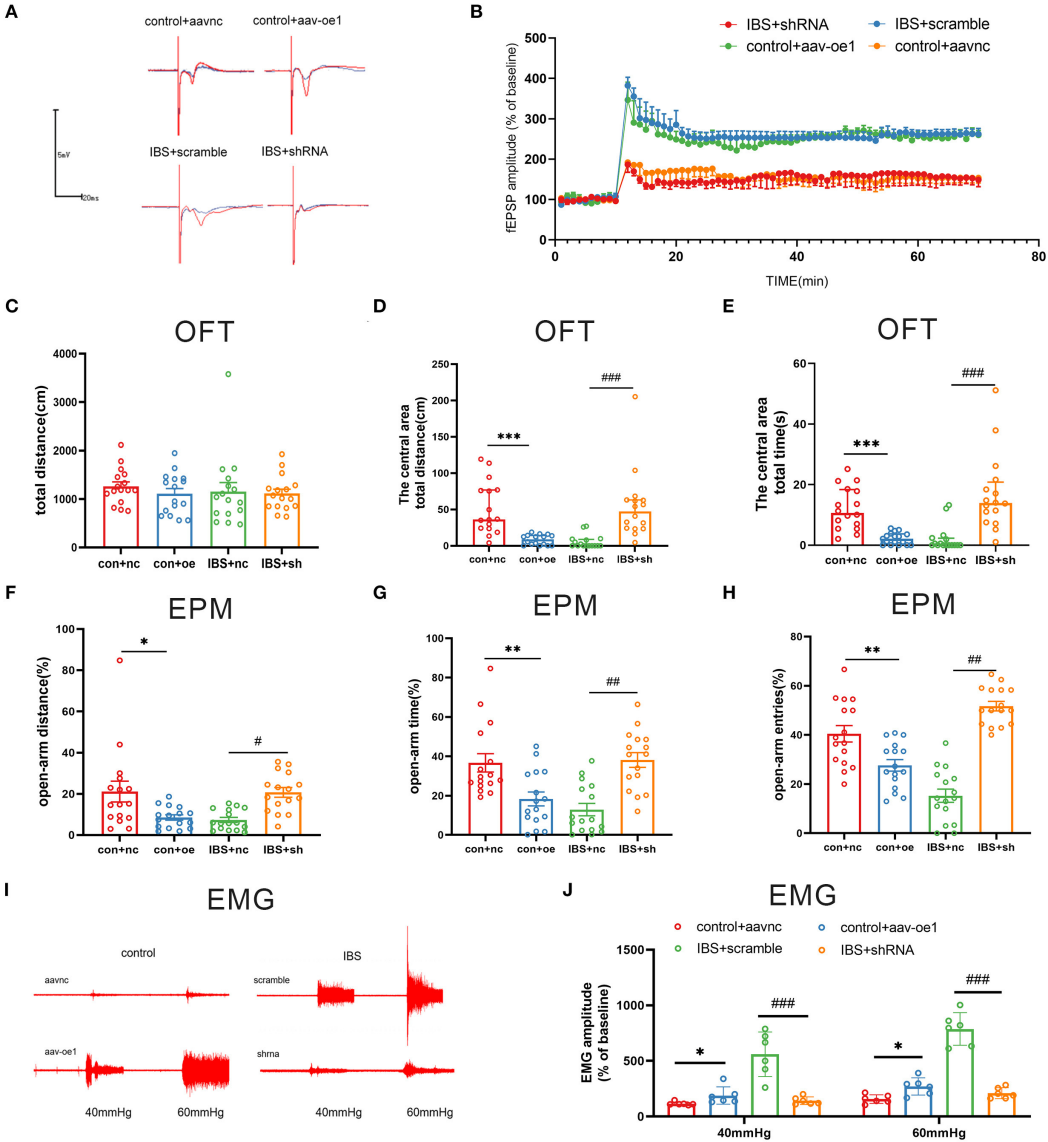
CA1 shcirckcnk9 attenuates LTP, anxiety and visceral hypersensitivity in IBS-like rats while overexpression of CA1 circKcnk9 induces LTP, anxiety and visceral hypersensitivity in controls. **(A,B)** Sample waveforms and summary bar charts of field potential before and 60 min after HFS in the slices of hippocampus. *N* = 3, *p < 0.05. Statistical analysis of the total distance **(C)**, the distance **(D)** and time **(E)** in the central area of the OFT. *N* = 16, ***p < 0.001, ^###^*p* < 0.001. Statistical analysis of the distance **(F)**, the time **(G)**, and the number of entries **(H)** in the open arm of EPM. *N* = 16, *p < 0.05, **p < 0.01, ^#^*p* < 0.05, ^##^*p* < 0.01. **(I)** The original typical recordings of EMG at 40 and 60 mmHg CRD. **(J)** The statistical chart of the percentage of EMG amplitude over baseline. *N* = 6, *P < 0.05, ^###^*p* < 0.001. CRD, colorectal distension; EMG, electromyography; EPM, elevated plus maze test; HFS, high-frequency stimulation; IBS, irritable bowel syndrome; LTP, long-term potentiation; OFT, open field test.

Since the expression of CA1 circKcnk9 increased in IBS-like rats, we observed the effects of the intervention at the CA1 circKcnk9 expression level on LTP, visceral pain, and anxiety. Adeno-associated virus (AAV) was microinjected into the CA1 of control rats to overexpress circKcnk9, whereas shcircKcnk9 was microinjected into the CA1 of IBS-like rats to knockdown circKcnk9. IF and qPCR assays confirmed that the circKcnk9 AAV/shRNA virus was successfully expressed in the hippocampus (Figures 2E,F). Field potential experiments revealed that overexpression of circKcnk9 enhanced CA1 LTP in hippocampal slices of control rats, whereas circKcnk9 knockdown attenuated CA1 LTP in IBS-like rats (Figures 3A,B). Compared with the controls with the empty-loaded virus, the control rats that overexpressed circKcnk9 traveled less time and distance in the central area of the OFT (Figures 3C–E) and had a decreased percentage of distance, time, and entries in the open-arm of the EPM (Figures 3F–H). The EMG results showed that circKcnk9 overexpression induced visceral hypersensitivity in the controls (Figures 3I,J). In contrast, IBS-like rats with CA1 shcircKcnk9 showed an increase in central distance and time in the OFT (Figures 3C–E) and had an increased percentage of distance, time, and entries in the open-arm of the EPM (Figures 3F–H). The EMG results also showed that CA1 shcircKcnk9 treatment alleviated visceral hypersensitivity in the IBS-like rats (Figures 3I,J).

Taken together, increased circKcnk9 was expressed in CA1 of IBS-like rats, and CA1 shcircKcnk9 treatment inhibited LTP and alleviated visceral hypersensitivity and anxiety in IBS-like rats. In contrast, overexpression of CA1 circKcnk9 enhanced LTP and induced visceral hypersensitivity and anxiety in control rats. Accordingly, we inferred that increased CA1 circKcnk9 expression induced by early life stress regulates visceral pain and anxiety by enhancing LTP in IBS-like rats.

### CircKcnk9 acts as a miR-124-3p sponge to regulate LTP, visceral hypersensitivity, and anxiety comorbidity in the CA1 of rats

**Figure 4:**
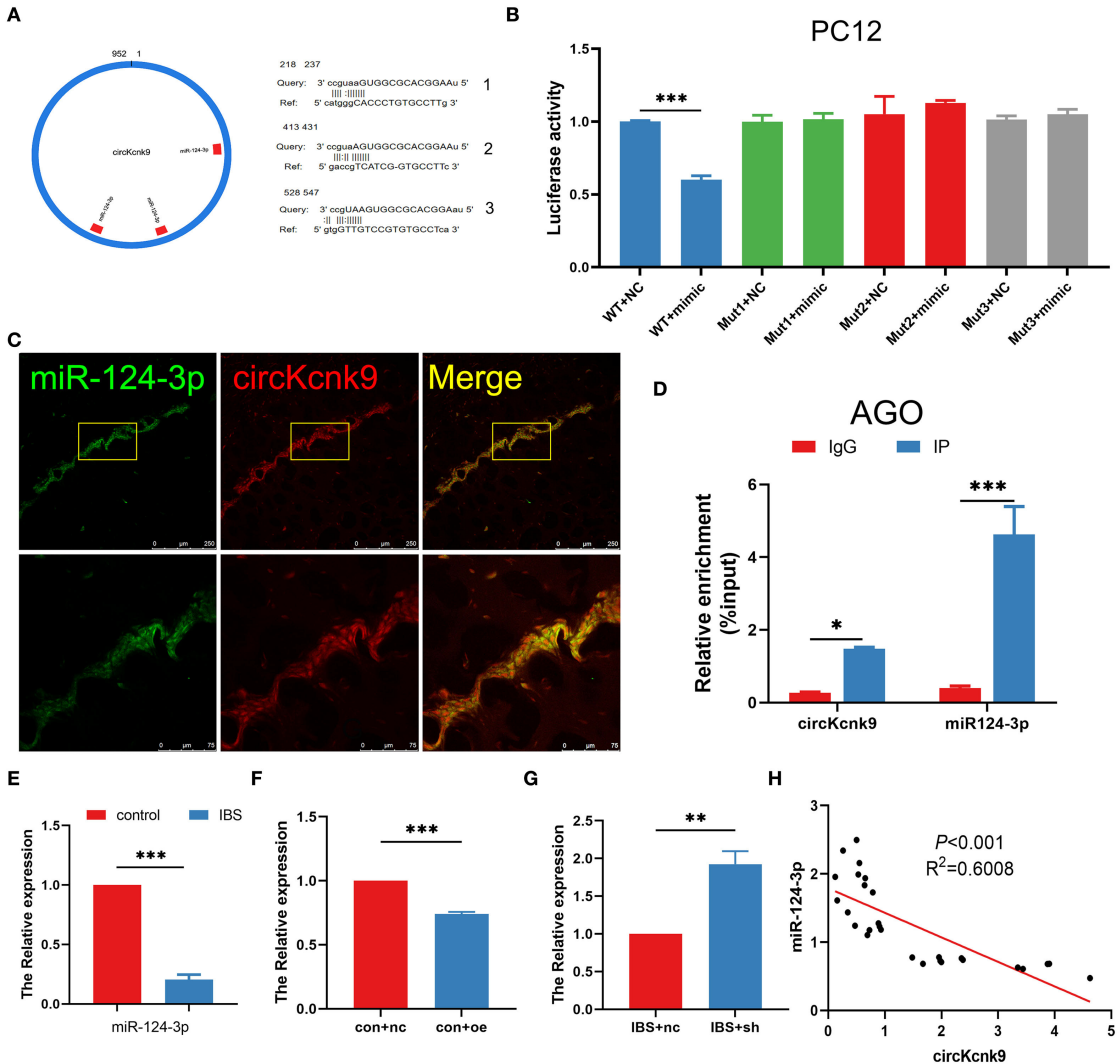
CircKcnk9 acts as miR-124-3p sponge. **(A)** CircKcnk9 has three binding sites for miR-124-3p. **(B)** Double-luciferase reporter assay results showing circKcnk9 could bind to miR-124-3p. *N* = 3, ***p < 0.001. **(C)** Colocalization of circKcnk9 and miR-124-3p. Scale bar, 250μm. **(D)** AGO protein antibodies can coprecipitate circKcnk9 and miR-124-3p. *N* = 4, *p < 0.05, ***p < 0.001. **(E)** Lower expression of hippocampal miR-124-3p in IBS rats compared with that in controls. *N* = 8, ***P < 0.001. Expression of miR-124-3p after CA1 treatment with aav-circKcnk9 in controls **(F)** and shcircKcnk9 in IBS-like rats **(G)**. *N* = 6, **p < 0.01, ***p < 0.001. (**H**) Correlation analysis of circKcnk9 and miR-124-3p (*p* < 0.001, *R*^2^ =0.6008). AGO: the vital component of the RNA-induced silencing complex.

The literature reports that circRNAs could act as miRNA sponges to modulate the expression of target genes (Kristensen et al., 2019; Van Zonneveld et al., 2021). We, therefore, used the miRanda database to predict the binding sites and found that circKcnk9 had three miR-124-3p binding sites (Figure 4A). The dual-luciferase reporter assay suggested that circKcnk9 could directly adsorb miR-124-3p through three predicted sites (Figure 4B). Confocal images confirmed the colocalization of circKcnk9 with miR-124-3p (Figure 4C). The RIP assay showed that circKcnk9 coprecipitated with miR-124-3p via AGO2, a vital component of the RNA-induced silencing complex, indicating that circKcnk9 acted as a miR-124-3p sponge (Figure 4D). qPCR showed that hippocampal miR-124-3p expression decreased in IBS-like rats (Figure 4E). In addition, qPCR results suggested that CA1 circKcnk9 overexpression reduced the expression of miR-124-3p in control rats (Figure 4F), whereas CA1 shcircKcnk9 treatment increased miR-124-3p expression in IBS-like rats (Figure 4G), indicating a negative correlation between circKcnk9 and miR-124-3p, which was further confirmed by correlation analysis (Figure 4H).

**Figure 5:**
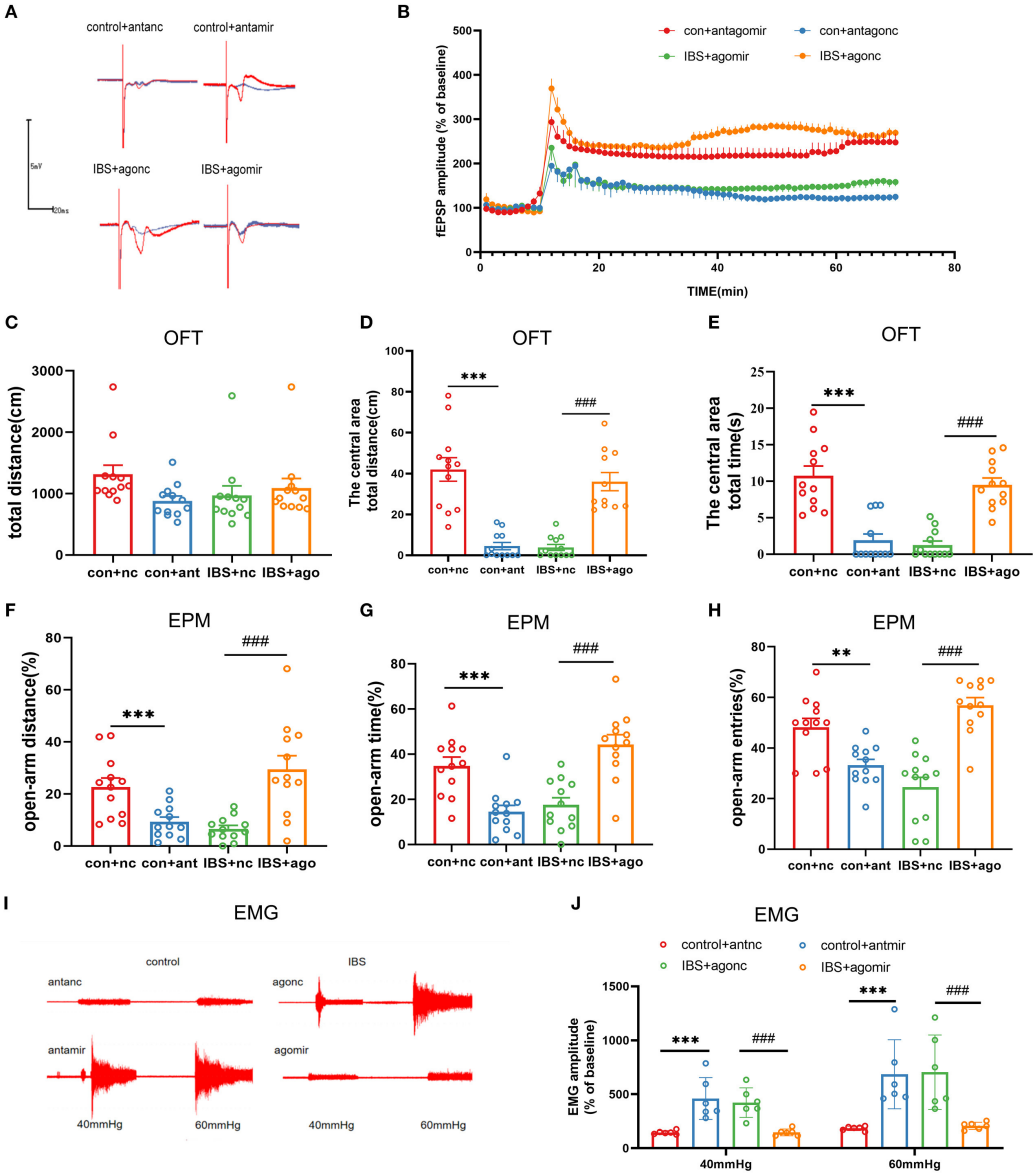
CA1 miR-124-3p upregulation decreased LTP, visceral hypersensitivity and anxiety in IBS-like rats while miR-124-3p blockade induced LTP, visceral hypersensitivity and anxiety in control rats. **(A,B)** Sample waveforms and summary bar charts of field potential before and 60 min after HFS in the slices of hippocampus. *N* = 3, *P < 0.05. Statistical analysis of the total distance **(C)**, the distance **(D)** and time **(E)** in the central area of the OFT. *N* = 12, ***p < 0.001, ^###^*p* < 0.001. Statistical analysis of the distance **(F)**, the time **(G)**, and the number of entries **(H)** in the open arm of EPM. *N* = 12, **p < 0.01, ***p < 0.001, ^###^*p* < 0.001. **(I)** The original typical recordings of EMG at 40 and 60 mmHg CRD. **(J)** The statistical chart of the percentage of EMG amplitude over baseline. *N* = 6, ***p < 0.001, ^###^*p* < 0.001. CRD, colorectal distension; EMG, electromyography; EPM, elevated plus maze test; HFS, high-frequency stimulation; IBS, irritable bowel syndrome; LTP, long-term potentiation; OFT, open field test.

As the expression of CA1 miR-124-3p decreased in IBS-like rats, we examined the influence of modifying CA1 miR-124-3p expression on LTP, visceral pain, and anxiety in rats. AntagomiR-124-3p was microinjected into the CA1 of control rats to inhibit the expression of miR-124-3p, whereas agomiR-124-3p was microinjected into the CA1 of IBS-like rats to increase the expression of miR-124-3p. Field potential experiments showed that inhibiting miR-124-3p enhanced CA1 LTP in the hippocampal slices of control rats, whereas increasing miR-124-3p attenuated CA1 LTP in IBS-like rats (Figures 5A,B). Compared with the antagomiR-NC group, the rats treated with antagomiR-124-3p in the CA1 traveled less time and distance in the central area of the OFT (Figures 5C–E) and had a decreased percentage of distance, time, and entries in the open-arm of the EPM (Figures 5F–H). The EMG results showed that miR-124-3p inhibition induced visceral hypersensitivity in the controls (Figures 5I,J). In contrast, IBS-like rats with CA1 administration of agomiR-124-3p showed an increase in the central distance and time in the OFT (Figures 5C–E) and had an increased percentage of distance, time, and entries in the open-arm of the EPM (Figures 5F–H). The EMG results also showed that CA1 treatment with agomiR-124-3p alleviated visceral hypersensitivity in IBS-like rats (Figures 5I,J). These results indicated that increased CA1 circKcnk9 acted as a miR-124-3p sponge, which resulted in the inhibiting effect of miR-124-3p on gene silencing to cause enhanced LTP visceral hypersensitivity and anxiety in IBS-like rats.

### EZH2 is a target gene of CircKcnk9/miR-124-3p and affects visceral hypersensitivity and anxiety in IBS-like rats by modifying CA1 LTP.

**Figure 6:**
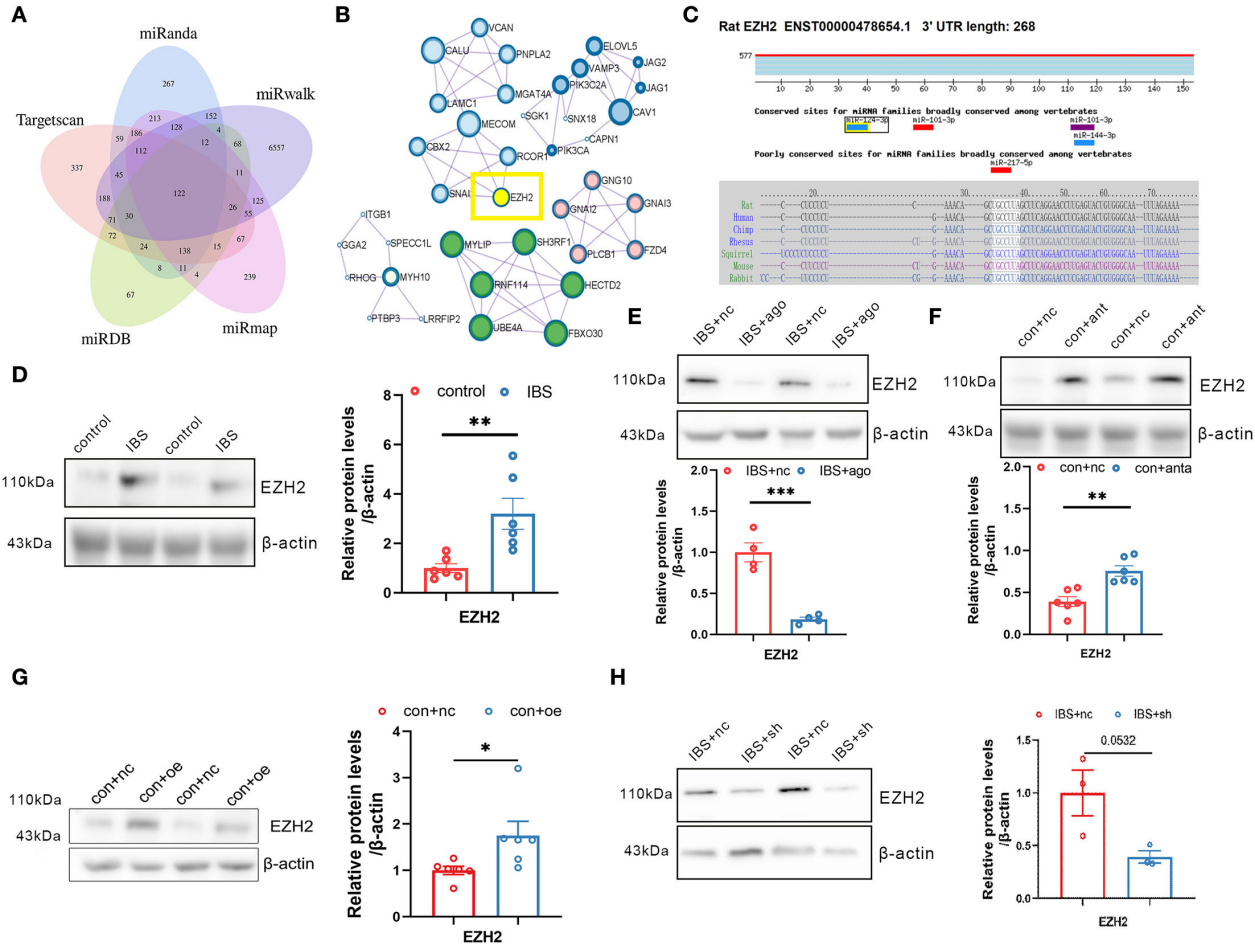
Hippocampal EZH2 is regulated by circKcnk9 and miR-124-3p. **(A)** Interactions between miR-124-3p and its target genes were predicted using Target Scan, miRDB, miRwalk, miRmap and miRanda databases. **(B)** MCODE in Cytoscape candidate target gene PPI network. **(C)** Sequence conservation of EZH2 binding site for miR-124-3p. **(D)** Representative images **(left)** and quantification **(right)** of the EZH2 protein expression in hippocampus of control and IBS-like rats. *N* = 6, **p < 0.01. Western blot quantification of EZH2 after CA1 treatment of agomiR-124-3p in IBS-like rats (**E**; *N* = 4) and antagomir in controls (**F;** *N* = 6). **p < 0.01, ***p < 0.001. Western blot quantification of EZH2 after CA1 treatment of aav-circKcnk9 in control (**G;** *N* = 6) and shcircKcnk9 in IBS-like rats (**H;** *N* = 3). *p < 0.05. MCODE, MCODE network clustering analysis; PPI, protein-protein interaction.

Because a single miRNA has the potential to regulate hundreds of transcripts, we predicted putative targets for miR-124-3p using five databases: miRanda, Targetscan, miRDB, miRmap, and miRwalk (Figure 6A). We used the Molecular Complex Detection (MCODE) algorithm for multiple target genes of miR-124-3p and screened the HUB genes (Figure 6B). EZH2 was selected as the target gene of miR-124-3p in IBS-like rats because EZH2 is associated with anxiety and pain. Bioinformatic analysis showed that the binding sequence of EZH2 with miR124-3p was highly conservative in humans, mice, and rats (Figure 6C). Western blotting showed a significant increase in CA1 EZH2 expression in IBS-like rats (Figure 6D). Furthermore, the expression of EZH2 decreased after administration of agomiR-124-3p in IBS-like rats (Figure 6E), whereas the expression of EZH2 increased after antagomiR-124-3p administration in controls (Figure 6F), indicating that miR-124-3p could inhibit EZH2 expression in the hippocampus. Furthermore, we speculated that circKcnk9 regulated CA1 EZH2 expression because we found that increased CA1 circKcnk9 acted as a miR-124-3p sponge in IBS-like rats. Therefore, we examined the protein expression of EZH2 after intervening at the level of circKcnk9. Overexpression of CA1 circKcnk9 increased the protein level of EZH2 in control rats (Figure 6G), whereas CA1 shcircKcnk9 decreased the protein level of EZH2 in IBS-like rats (Figure 6H).

**Figure 7:**
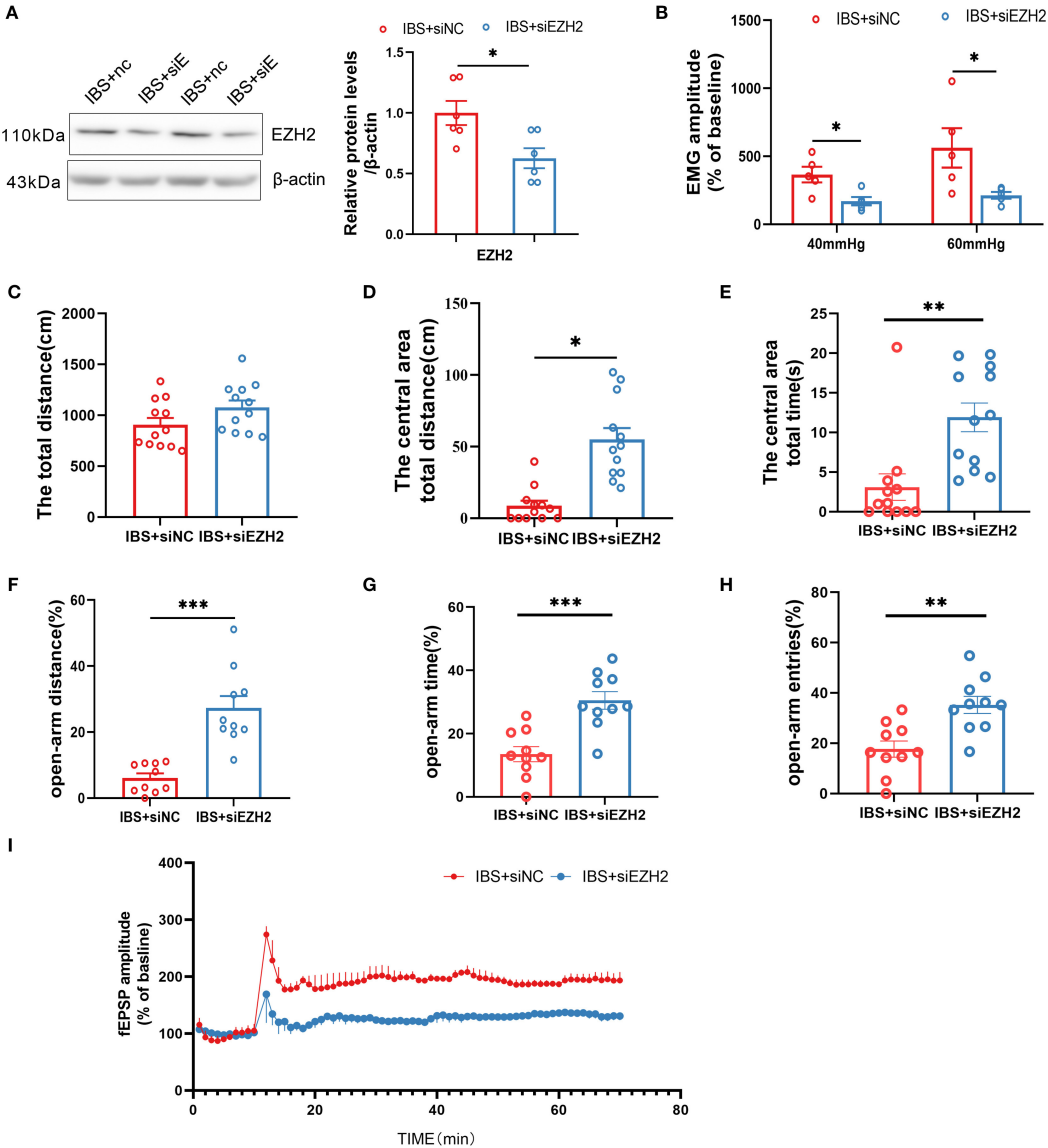
Downregulation of CA1 EZH2 attenuates anxiety, visceral hypersensitivity and LTP in IBS-like rats. **(A)** EZH2 protein expression decreased after administration of siEZH2 into CA1 in IBS-like rats. *N* = 6, *p < 0.05. **(B)** The statistical chart of the percentage of EMG amplitude over baseline. *N* = 5, *p < 0.05. Statistical analysis of the total distance **(C)**, the distance **(D)** and time **(E)** in the central area of the OFT. *N* = 12, *p < 0.05, **p < 0.01. Statistical analysis of the distance **(F)**, the time **(G)**, and the number of entries **(H)** in the open arm of EPM. *N* = 10, **p < 0.01, ***p < 0.001. **(I)** Summary bar charts of field potential before and 60 min after HFS in the slices of hippocampus. *N* = 3, *p < 0.05. EMG, electromyography; EPM, elevated plus maze test; HFS, High frequency stimulation; IBS, irritable bowel syndrome; OFT, open field test.

In light of our finding that CA1 EZH2 expression was increased in IBS-like rats, we microinjected siEZH2 into CA1 to knock down EZH2 and examine its effects on visceral hypersensitivity, anxiety, and CA1 LTP. Western blotting confirmed that CA1 EZH2 expression decreased after CA1 treatment with siEZH2 in IBS-like rats (Figure 7A). The EMG results showed that administration of siEZH2 into CA1 alleviated visceral hypersensitivity in the IBS-like rats (Figure 7B). IBS-like rats with CA1 treatment of siEZH2 showed an increase in central distance and time in the OFT (Figures 7C–E) and had an increased percentage of distance, time, and entries in the open arm of the EPM (Figures 7F–H). In addition, CA1 treatment with siEZH2 attenuated CA1 LTP in the hippocampal slices of IBS-like rats (Figure 7I). These results suggested that EZH2 was a target gene of miR-124-3p and that increased EZH2 expression was involved in visceral hypersensitivity and anxiety in IBS-like rats by regulating synaptic plasticity.

## Discussion

This study explored the epigenetic molecular mechanisms underlying visceral hypersensitivity and anxiety comorbidities in IBS-like rats. Neonatal CRD-induced visceral hypersensitivity and anxiety in IBS-like rats. The expression of hippocampal circKcnk9, a novel circRNA, was significantly increased in IBS-like rats. CA1 shcircKcnk9 treatment inhibited LTP and alleviated visceral hypersensitivity and anxiety in IBS-like rats, whereas overexpression of CA1 circKcnk9 enhanced LTP and induced visceral hypersensitivity and anxiety in control rats. Several experiments indicate that increased CA1 circKcnk9 acts as a miR-124-3p sponge, inhibiting the role of miR-124-3p in gene silencing from causing enhanced LTP, visceral hypersensitivity, and anxiety in IBS-like rats. CA1 administration of agomiR-124-3p decreased CA1 LTP amplitude, visceral pain response, and anxiety in IBS-like rats. In contrast, CA1 treatment with antagomir-124-3p induced LTP, visceral hypersensitivity, and anxiety in the control rats. Furthermore, bioinformatics analysis and experimental data showed that EZH2 is a target gene of miR-124-3p and that increased EZH2 expression is involved in visceral hypersensitivity and anxiety in IBS-like rats by regulating synaptic plasticity.

The current study shows neonatal CRD-induced visceral hypersensitivity and anxiety in IBS-like rats. Our previous study demonstrated that LTP is enhanced in the hippocampal CA1 region of IBS-like rats (Chen et al., 2014). As a model of early life stress, neonatal CRD may induce abnormal synaptic plasticity and adverse memory in the hippocampus, which is at least partly responsible for visceral hypersensitivity and anxiety comorbidity in IBS-like rats.

Chronic phenotypes are often associated with the epigenetic modulation of gene expression. In light of the literature suggesting that hippocampal circRNAs may be involved in the regulation of pain (Zheng et al., 2022), negative emotions (Huang et al., 2020), and synaptic plasticity (Xu et al., 2020), we compared the differential expression of several circRNAs in the hippocampal CA1 between IBS-like rats and controls. Interestingly, we found that CA1 circKcnk9 was significantly increased in IBS-like rats. Moreover, circKcnk9 is largely colocalized with neurons but not microglia or astrocytes, which is supported by studies showing that circRNAs are abundantly expressed in neurons (You et al., 2015; Knupp et al., 2021). Different circRNAs may play different roles in chronic pain and negative emotions. For example, circ-Ankib1 and circAnks1a are involved in the development of neuropathic pain (Zhang S. B. et al., 2019); circRNA_104670 and circSEMA4B are involved in low back pain (Lin et al., 2021); circSlc7a11 overexpression is involved in bone cancer pain (Chen et al., 2021); and circSTAG1 was significantly decreased in the hippocampus of chronic unpredictable stress-treated mice and the peripheral blood of patients with major depressive disorder (Huang et al., 2020). These studies indicate that the roles of different circRNAs are relatively specific and could potentially be used for disease diagnosis and treatment. However, the role of circKcnk9 in other disorders has not been reported.

Because of the increased expression of CA1 circKcnk9 in IBS-like rats, we hypothesized that intervention in the expression level of CA1 circKcnk9 could regulate LTP, visceral pain, and anxiety in rats. In support of this, AAV was microinjected into the CA1 of control rats to overexpress circKcnk9, while shcircKcnk9 was microinjected into the CA1 of IBS-like rats to knockdown circKcnk9. As expected, overexpression of hippocampal circKcnk9 induced visceral hypersensitivity and anxiety in controls, whereas knockdown of circkcnk9 attenuated visceral hypersensitivity and anxiety in IBS-like rats. Behavioral evidence supported our finding that the CA1 circKcnk9 is a key molecule for visceral hypersensitivity and anxiety comorbidity in IBS-like rats. In line with the behavioral results, electrophysiological experiments revealed that overexpression of CA1 circKcnk9 facilitated high-frequency-induced LTP in controls, whereas knockdown of circkcnk9 inhibited LTP in IBS-like rats, indicating that circKcnk9 could affect synaptic plasticity and cause central sensitivity. Furthermore, it was reported that neural circRNAs are derived from synaptic genes and are regulated by development and plasticity (You et al., 2015), which is consistent with our findings. Taken together, our results suggest that neonatal CRD stress induces increased CA1 circKcnk9 expression, leading to visceral hypersensitivity and anxiety comorbidity by regulating synaptic plasticity in IBS-like rats.

Studies have reported that circRNAs can act as miRNA sponges to modulate the expression of target genes (Zeng et al., 2021). Chronic pain-related studies found that miR-124-3p is involved in the neuropathic pain of CCI rat models (Zhang Y. et al., 2019; Wei et al., 2020). In addition, mice exposed to chronic ultra-mild stress exhibited increased depression-like behaviors and reduced hippocampal expression of brain-enriched miR-124 (Higuchi et al., 2016). Our results showed that increased CA1 circKcnk9 expression led to visceral hypersensitivity and anxiety in IBS-like rats. We next sought to explore the mechanism underlying the role of CA1 circKcnk9 in IBS-like rats. Confocal images and a RIP assay confirmed that circKcnk9 colocalized with miR-124-3p and acted as a miR-124-3p sponge.

Furthermore, the miRanda database and dual-luciferase reporter assay suggested that circKcnk9 could directly adsorb miR-124-3p through three predicted sites. Meanwhile, qPCR quantification showed that hippocampal miR-124-3p expression was decreased in IBS-like rats. Since circKcnk9 acts as a miR-124-3p sponge, there is a negative correlation between circKcnk9 and miR-124-3p. It is conceivable that the gene regulation of CA1 miR-124-3p will have opposite effects on CA1 LTP and behavior in rats. Electrophysiological and behavioral experiments confirmed this speculation that CA1 miR-124-3p upregulation decreased CA1 LTP amplitude, visceral hypersensitivity, and anxiety in IBS-like rats, while miR-124-3p downregulation induced LTP, visceral hypersensitivity, and anxiety in control rats. Intrahippocampal supplementation with miR-124-3p could be a novel therapy for IBS, which has also been studied in epilepsy (Wang et al., 2016).

MicroRNAs are important post-transcriptional regulators of gene expression that act by direct base pairing to target sites within the untranslated regions of mRNAs. Studies have reported that the miR-124-3p/EZH2 signaling pathway is involved in cancer (Sha et al., 2020; Yang et al., 2021; Zhu et al., 2021). MiR-124-3p was also found to be significantly downregulated in rats after chronic sciatic nerve injury, and its direct target gene was EZH2 (Zhang Y. et al., 2019). To explore whether EZH2 is a target gene of circKcnk9/miR-124-3p in the hippocampus of IBS-like rats, we designed several experiments to clarify the molecular mechanism. We screened EZH2 through bioinformatics analysis and confirmed that EZH2 is one of the target genes for miR-124-3p, and the binding sequence of EZH2 with miR-124-3p is highly conservative in humans, mice, and rats. Moreover, western blotting showed a significant increase in CA1 EZH2 expression in IBS-like rats. Furthermore, the expression of EZH2 decreased after the upregulation of miR-124-3p or downregulation of circKcnk9 in the CA1 of IBS-like rats. Then, through stereotaxic administration of siEZH2 into CA1 to knock down EZH2, its effects on visceral hypersensitivity, anxiety, and CA1 LTP were examined. CA1 treatment with siEZH2 downregulated CA1 LTP, visceral hypersensitivity, and anxiety in IBS-like rats. EZH2 is a target gene of miR-124-3p and other miRNAs (Li et al., 2020; Gao P. et al., 2021; Zhang et al., 2021). It is reasonable to consider that EZH2 is the common cellular pathway involved in many disorders, including cancer (Anwar et al., 2018), chronic pain (Yadav and Weng, 2017), and negative emotions (Yan et al., 2019; Li et al., 2022).

## Conclusion

In conclusion, the most important finding of this study is that early life stress induces increased expression of circKcnk9, a novel circRNA, in the CA1 of IBS-like rats. Increased circKcnk9 expression regulates synaptic transmission and enhances LTP, leading to visceral hypersensitivity and anxiety in IBS-like rats. The underlying signaling pathway of circKcnk9 is miR-124-3p/EZH2. Increased circKcnk9 reinforces its sponge for miR-124-3p, strongly suppressing miR-124-3p action and resulting in increased expression of the target gene EZH2. This study provides a new epigenetic mechanism of visceral hypersensitivity and anxiety comorbidity in IBS-like rats, and circKcnk9 may be a key molecule in the treatment of IBS.

## Data availability statement

The original contributions presented in the study are included in the article/Supplementary material, further inquiries can be directed to the corresponding authors.

## Ethics statement

The animal study was reviewed and approved by the Animal Care and Use Committee of Fujian Medical University.

## Author contributions

YL and CL wrote the first draft and designed the research. YL, WL, ZL, and YZ performed the experiments. YC, ZC, YZ, and BW contributed to data acquisition. YL performed the bioinformatic analysis. BW, ZC, and YZ analyzed the data. YL, WL, CL, and AC wrote and revised the manuscript. All authors contributed to the article and approved the submitted version.

## Conflict of interest

The authors declare that the research was conducted in the absence of any commercial or financial relationships that could be construed as a potential conflict of interest.

## Publisher's note

All claims expressed in this article are solely those of the authors and do not necessarily represent those of their affiliated organizations, or those of the publisher, the editors and the reviewers. Any product that may be evaluated in this article, or claim that may be made by its manufacturer, is not guaranteed or endorsed by the publisher.
