# Supplementary figures and images for: Role of hippocampal circKcnk9 in visceral hypersensitivity and anxiety comorbidity of irritable bowel syndrome
Source: Front Cell Neurosci. 2022 Nov 17;16:1010107. doi: 10.3389/fncel.2022.1010107 (PMC9714028; doi:10.3389/fncel.2022.1010107)

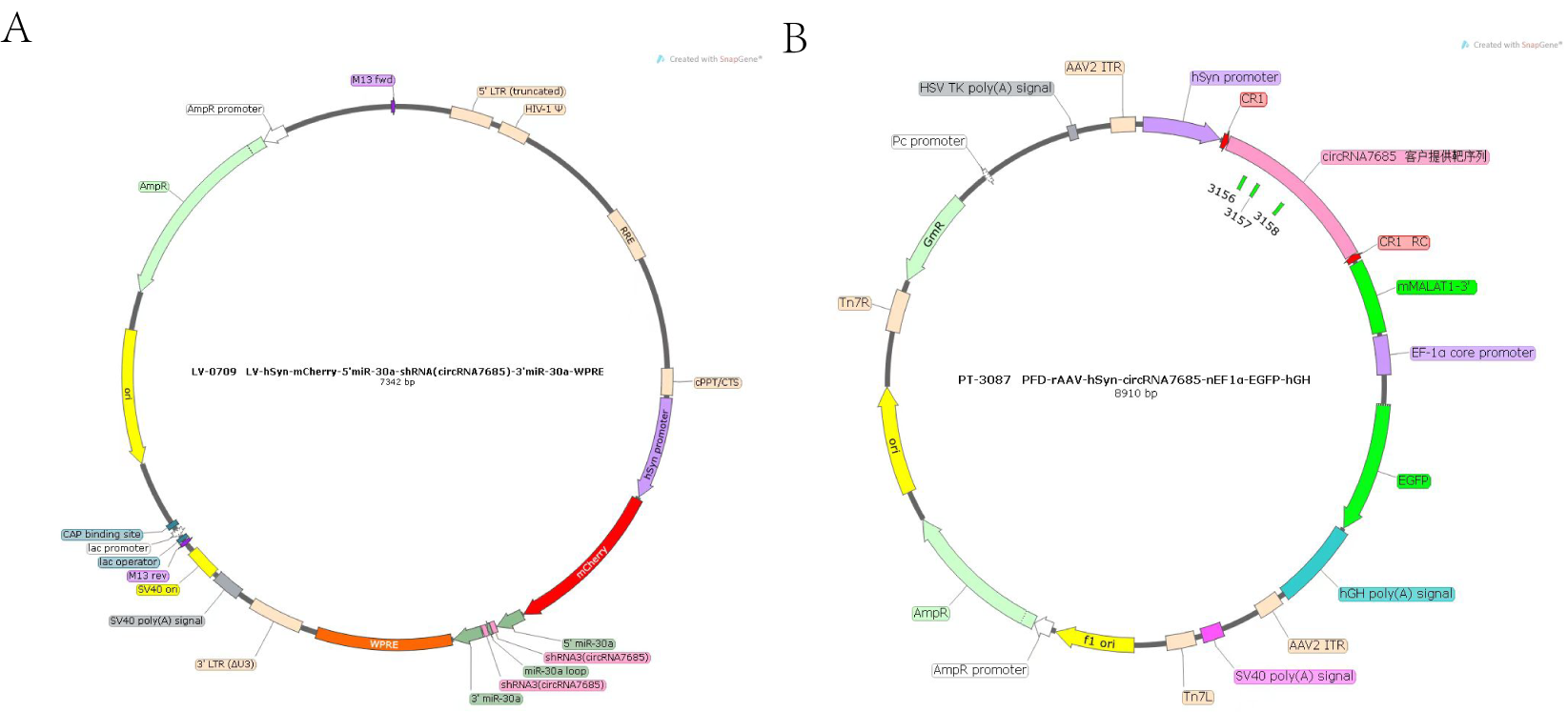

Supplement: Supplementary file 1 [file Image_1.tif]

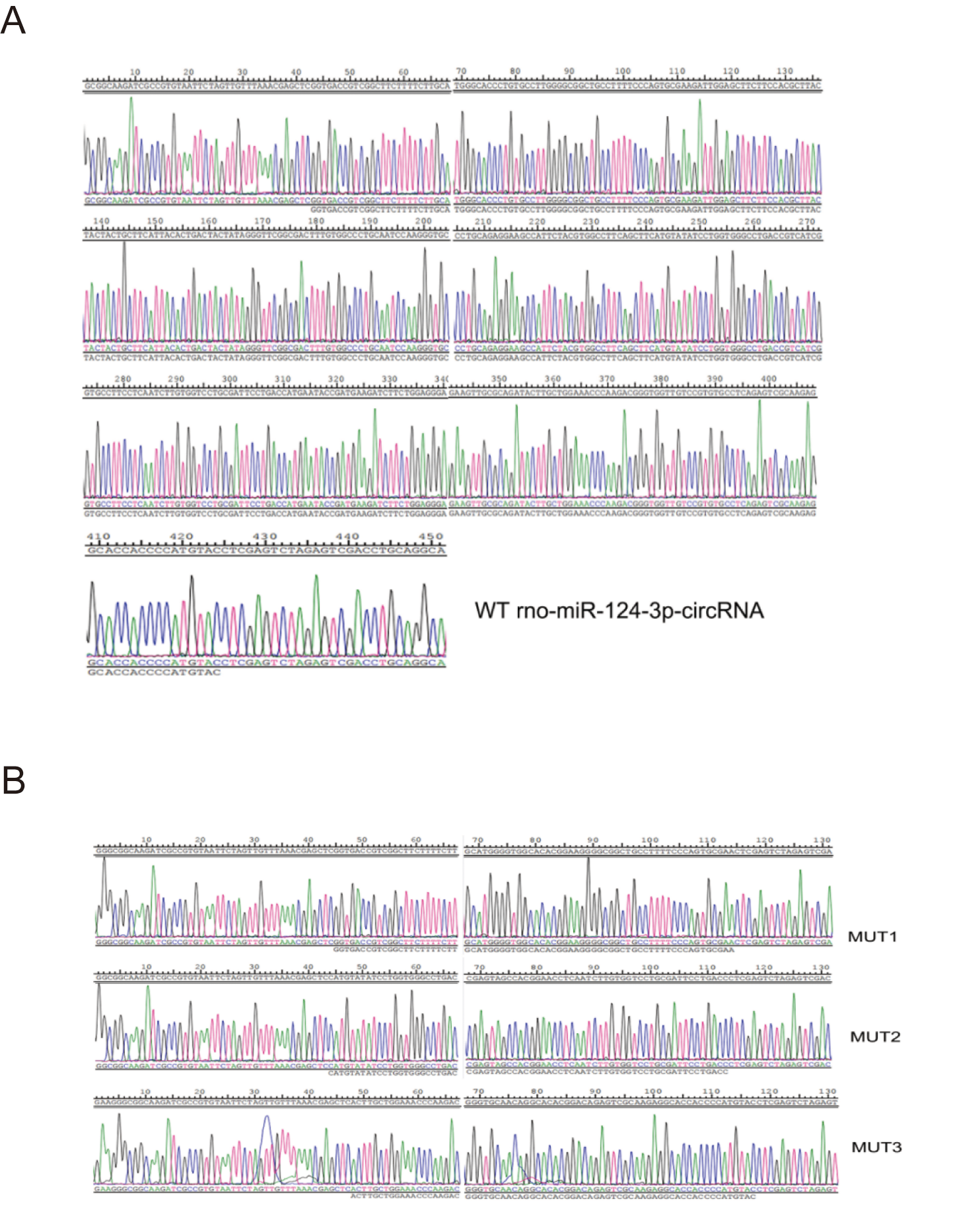

Supplement: Supplementary file 2 [file Image_2.tif]
